# Manganese uptake by MtsABC contributes to the pathogenesis of human pathogen group A streptococcus by resisting host nutritional immune defenses

**DOI:** 10.1128/iai.00077-24

**Published:** 2024-06-13

**Authors:** Nishanth Makthal, Subhasree Saha, Elaine Huang, Juliane John, Himani Meena, Shifu Aggarwal, Martin Högbom, Muthiah Kumaraswami

**Affiliations:** 1 Center for Molecular and Translational Human Infectious Diseases Research, Houston Methodist Research Institute, Houston, Texas, USA; 2 Department of Pathology and Genomic Medicine, Houston Methodist Hospital, Houston, Texas, USA; 3 Department of Biochemistry and Biophysics, Stockholm University, Arrhenius Laboratories for Natural Science, Stockholm, Sweden; University of Illinois Chicago, Chicago, Illinois, USA

**Keywords:** manganese, metal uptake, nutritional immunity, pathogenesis, streptococcus

## Abstract

The interplay between host nutritional immune mechanisms and bacterial nutrient uptake systems has a major impact on the disease outcome. The host immune factor calprotectin (CP) limits the availability of essential transition metals, such as manganese (Mn) and zinc (Zn), to control the growth of invading pathogens. We previously demonstrated that the competition between CP and the human pathogen group A streptococcus (GAS) for Zn impacts GAS pathogenesis. However, the contribution of Mn sequestration by CP in GAS infection control and the role of GAS Mn acquisition systems in overcoming host-imposed Mn limitation remain unknown. Using a combination of *in vitro* and *in vivo* studies, we show that GAS-encoded *mtsABC* is a Mn uptake system that aids bacterial evasion of CP-imposed Mn scarcity and promotes GAS virulence. Mn deficiency caused by either the inactivation of *mtsC* or CP also impaired the protective function of GAS-encoded Mn-dependent superoxide dismutase. Our *ex vivo* studies using human saliva show that saliva is a Mn-scant body fluid, and Mn acquisition by MtsABC is critical for GAS survival in human saliva. Finally, animal infection studies using wild-type (WT) and *CP−/*− mice showed that MtsABC is critical for GAS virulence in WT mice but dispensable in mice lacking CP, indicating the direct interplay between MtsABC and CP *in vivo*. Together, our studies elucidate the role of the Mn import system in GAS evasion of host-imposed metal sequestration and underscore the translational potential of MtsABC as a therapeutic or prophylactic target.

## INTRODUCTION

Transition metals, such as iron (Fe), manganese (Mn), and zinc (Zn), are cofactors for various metalloproteins that are involved in critical bacterial pathophysiological processes (1–3). Thus, pathogenic bacteria require Fe, Mn, and Zn to survive and establish infection in the host. Consequently, the host evolved molecular strategies to sequester metals during infection and prevent the growth of invading pathogens. The host deploys nutritional immune factors, such as calprotectin (CP), to infection sites to withhold metals and inhibit microbial growth (4–8). However, bacterial pathogens possess adaptive mechanisms to evade host nutritional immune responses and survive in the host. The well-characterized bacterial counter strategies include high-affinity metal acquisition by bacterial surface-bound or secreted metal transport/scavenging systems, reduced metal usage by metal-sparing responses, and increased metal mobilization from intracellular stores (5, 6, 9–13). In accordance with their significance to bacterial survival in the host, the metal transporters are major virulence determinants of various human pathogens (9–14).

*Streptococcus pyogenes*, also known as group A streptococcus (GAS)*,* is an exclusive human pathogen that colonizes diverse host anatomic sites with varying nutrient availability and host immune mechanisms (15–18). GAS infections cause an array of disease manifestations ranging from mild pharyngitis and impetigo to life-threatening, severe invasive infections such as necrotizing fasciitis and streptococcal toxic shock syndrome (15–18). GAS-infected abscesses are enriched with neutrophil-derived host immune factor CP (13). CP, a heterodimer of S100A8 and S100A9 proteins, is recruited to the infection sites as a component of the first line of defenses by infiltrating neutrophils and participates in host defense against several bacterial pathogens, including GAS (5, 9, 11, 19–23). Secreted CP binds to calcium and sequesters extracellular Zn and Mn from bacterial colonization surfaces (6–8, 24). Metal binding in CP occurs at two sites that contribute to its antimicrobial activity: a Zn-binding site with a His_3_Asp motif, and a Zn/Mn site with a hexa-histidine motif (His_6_ motif) that chelates Zn or Mn. The His_3_Asp site is comprised of the amino acids H83 and H87 from S100A8 and H20 and D30 from S100A9, whereas the side chains of amino acids H17 and H27 from S100A8 and H91, H95, H103, and H105 from S100A9 coordinate metal binding at His_6_ site (6–8, 24).

Previously, we demonstrated that GAS encounters CP-dependent Zn limitation, which contributes to bacterial growth inhibition (13, 25). To overcome the Zn scarcity caused by CP, GAS upregulates the expression of genes involved in Zn acquisition, Zn storage, and Zn sparing (12). The genes involved in GAS adaptive responses to host-imposed Zn limitation are critical for cytosolic Zn homeostasis, CP resistance, and bacterial virulence in a mouse model of invasive infection (12). However, those genes are dispensable for GAS survival and virulence in CP-inactivated mice (12), indicating that GAS adaptive responses to Zn limitation play a direct role in evading CP-mediated Zn withholding in the host. Compared with the known contribution of GAS adaptation to Zn limitation to CP resistance, the impact of CP-mediated Mn limitation and genes involved in GAS adaptation to Mn limitation on bacterial virulence remain unknown.

**Fig 1:**
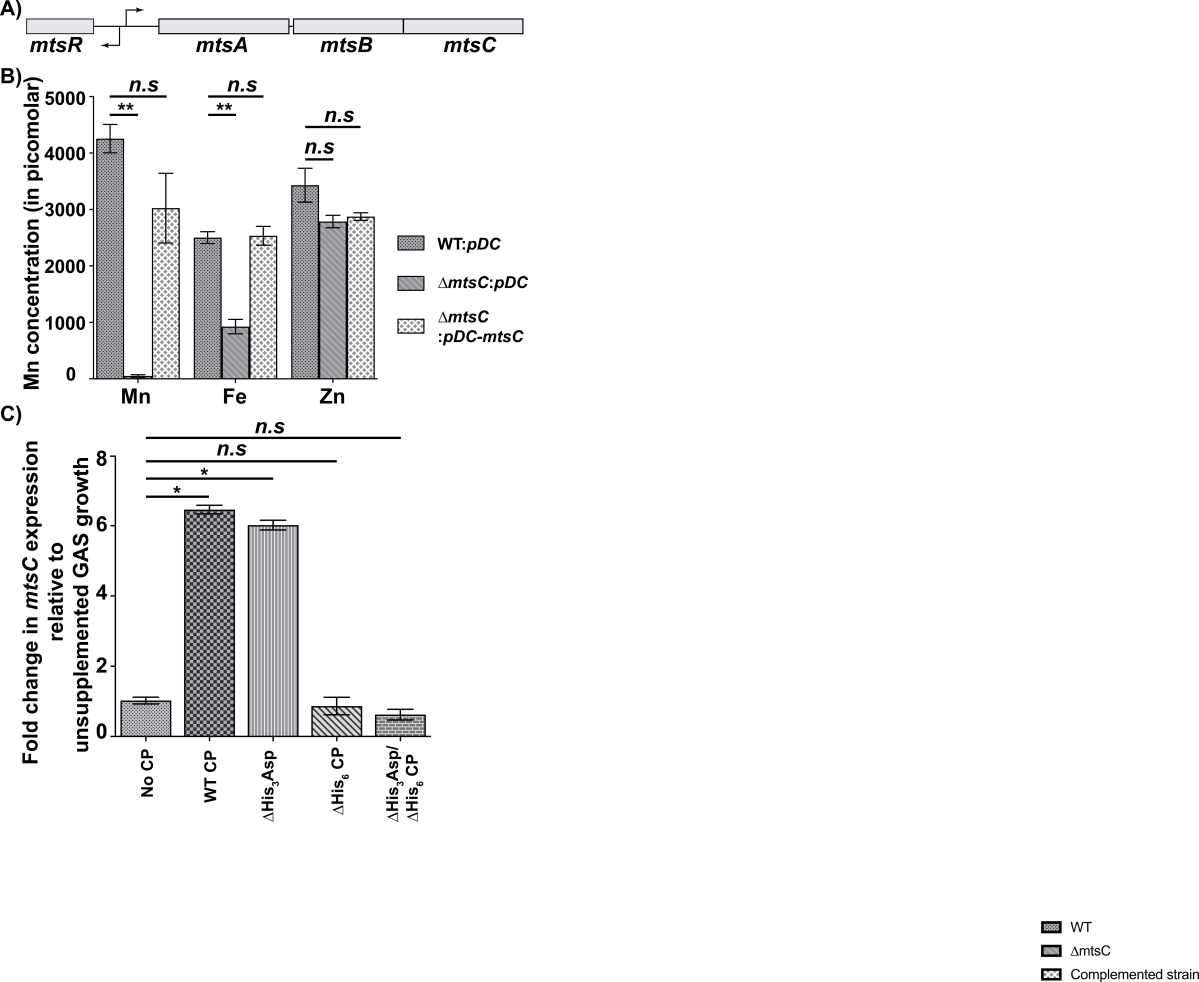
MtsABC is critical for GAS Mn acquisition. (**A**) Genetic organization of the operon-encoding *mtsABC* genes and the divergently transcribed *mtsR* gene encoding Mn-sensing transcription regulator in GAS genome is shown. The bent arrows above and below the line indicate the putative transcription start sites of *mtsABC* and *mtsR*, respectively. (**B**) Intracellular metal content of the indicated GAS strains as assessed using total-reflection X-ray fluorescence (TXRF) analyses. GAS strains were grown to mid-exponential growth phase (*A*_600_ 0.8) in Todd-Hewitt broth (THY) supplemented with 2 µM Mn (THY-M). Cell pellets were washed twice in phosphate-buffered saline (PBS) containing 1 mM nitrilotriacetic acid, followed by two washes in chelexed PBS, and suspended in chelexed PBS. The metal content in the clarified cell lysate was analyzed using TXRF analyses. Data graphed are mean ± standard deviation for three biological replicates. *P* values (**P < 0.01) were determined by comparison to the respective WT GAS control and were derived from the Kruskal–Wallis test. (**C**) WT GAS cells were grown to mid-exponential growth phase (*A*_600_ ~0.8) in the presence or absence of indicated concentrations of recombinant WT or mutant CP proteins. GAS grown in the absence of CP was used as the reference bacterial growth. Transcript levels of *mtsC* was assessed by reverse transcriptase quantitative PCR (qRT-PCR). The fold change in transcript levels relative to the reference is shown. Data graphed are mean ± standard deviation for three biological replicates grown on separate occasions. *P* values (**P < 0.01) were determined by comparison to GAS grown in the absence of CP and were derived from the Kruskal–Wallis test.

GAS primarily upregulates the genes encoding Mn importer *mtsABC* during growth in Mn-deficient conditions *in vitro* (14) and during colonization in the vaginal lumen (26). The tripartite Mn acquisition system comprises an extracellular metal-binding lipoprotein, MtsA, a cytosolic ATPase, MtsB, which provides the energy for metal import, and an inner membrane permease, MtsC (Fig. 1A). Previous studies have indicated that MtsA binds to iron with high affinity *in vitro,* and inactivation of *mtsABC* caused reduced cytosolic levels of Fe and Zn (27–29). However, recent data indicate a Mn-specific role for MtsABC as inactivation of *mtsABC* reduced intracellular Mn levels without affecting Fe levels (30). Thus, ambiguity exists regarding the specific metal transported by MtsABC. Nevertheless, MtsABC plays a critical role in GAS pathogenesis as the *mtsABC* mutant was sensitive to oxidative stress (30) and attenuated for virulence in invasive mouse models of infection (27, 30). However, the role of *mtsABC* in GAS defenses against host nutritional immune mechanisms *in vitro* and *in vivo* remain uncharacterized.

In this study, we investigated the role of MtsABC in the competition between CP and GAS for Mn during infection and its contribution to GAS pathogenesis. We show that Mn acquisition by MtsABC plays a crucial role in GAS evasion of CP-mediated Mn limitation and contributes to GAS virulence in a mouse model of invasive infection. However, MtsABC is dispensable for GAS pathogenesis in *CP−/*− mice, highlighting the significance of Mn in host–GAS interactions *in vivo* and the contribution of MtsABC to overcome the host-imposed Mn limitation during infection.

## RESULTS

### MtsABC is critical for Mn acquisition

To determine the role of MtsABC in metal acquisition by GAS, we measured the cytosolic metal levels of GAS grown in Mn-replete medium using total-reflection X-ray fluorescence (TXRF) analysis. To this end, we constructed an *mtsC*-inactivated mutant (∆*mtsC*) and a corresponding *trans*-complemented strain that was generated by introducing a plasmid (*pDC*) containing *mtsC* gene along with its native promoter into the ∆*mtsC* mutant (∆*mtsC:pDC-mtsC*) (Fig. S1). Transcript level analyses revealed that *trans*-complementation with *mtsC* restored WT-like *mtsC* expression in the ∆*mtsC* mutant (Fig. S1C). Since unmodified Todd–Hewitt broth (THY) has Mn at sub-micromolar levels (~250 nM) and mimics Mn-limiting growth conditions *in vitro* (14, 31), we used THY supplemented with 2 µM MnCl_2_ (THY-M) as the Mn replete medium. Cells grown to the mid-exponential phase of growth in THY-M were washed with chelexed buffer and lysed, and the clarified cell lysates were analyzed for intracellular metal content. Compared to WT, intracellular Mn levels were below detection limits in the ∆*mtsC* mutant, whereas the *trans*-complemented (∆*mtsC:pDC-mtsC*) strain had WT-like Mn levels (Fig. 1B). Similar to previous findings (27), the inactivation of *mtsC* also caused a significant reduction in cytosolic iron levels; however, it did not impact cytosolic Zn levels (Fig. 1B). These results show that MtsABC plays a major role in Mn and Fe acquisition by GAS.

### GAS employs *mtsABC* during growth in the presence of CP

GAS activates the expression of genes encoding Zn acquisition and sparing systems to overcome host nutritional immune mechanisms and survive in the host (12, 13). Since GAS upregulates *mtsABC* expression under Mn-limiting growth conditions *in vitro* (14), and MtsABC is involved in Mn acquisition (Fig. 1B), we hypothesized that GAS upregulates *mtsABC* expression during growth in the presence of CP to evade CP-mediated Mn limitation. To test this hypothesis, we compared *mtsC* transcript levels in GAS grown in the presence or absence of recombinant CP *in vitro* by reverse transcriptase quantitative PCR (qRT-PCR). A significant upregulation of *mtsC* was observed in the presence of WT CP compared to GAS grown in the absence of CP (Fig. 1C). To determine whether the induction of *mtsC* expression is specific for CP-mediated Mn limitation, we performed similar experiments with recombinant mutant CP proteins that are defective in either Zn (∆His_3_Asp site) or Zn/Mn (∆His_6_ site) binding. Furthermore, we included recombinant ∆His_3_Asp/∆His_6_ site mutant CP in which both Zn and Zn/Mn binding sites were inactivated. Our results show that GAS upregulated *mtsC* expression only in the presence of ∆His_3_Asp site mutant CP that is capable of imposing Mn limitation, whereas no induction of *mtsC* expression was observed in the presence of either recombinant ∆His_6_ site or ∆His_3_Asp/∆His_6_ site mutant CP that are defective in Mn binding (Fig. 1C). Collectively, these results indicate that GAS upregulates *mtsABC* specifically to combat CP-mediated Mn limitation.

### MtsABC is critical for GAS survival in the presence of CP

**Fig 2:**
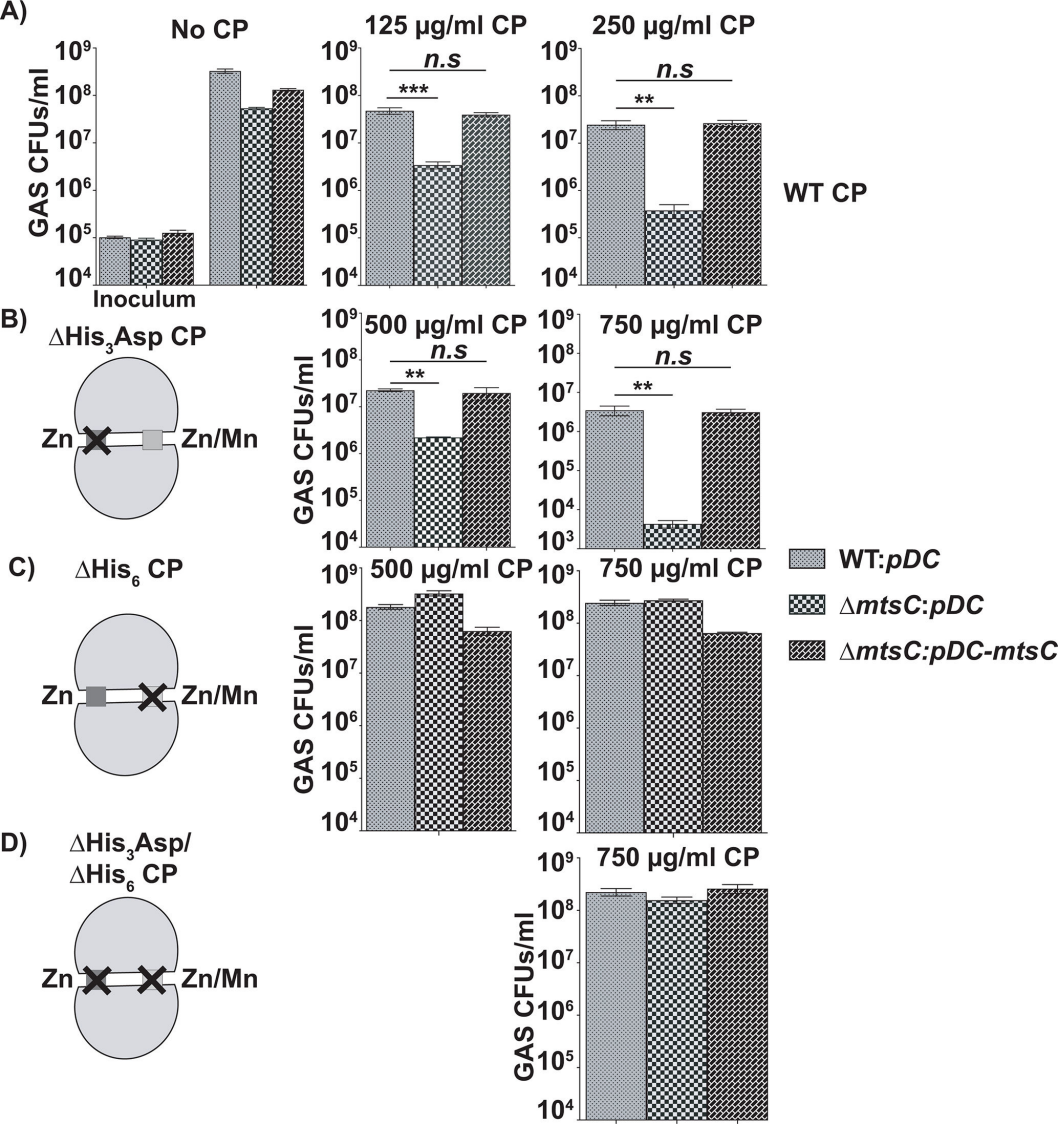
Gene encoding *mtsC* is critical for GAS defense against CP-mediated Mn limitation. The survival of GAS grown in the presence or absence of recombinant CP, as assessed using CFU analyses, is shown. GAS was inoculated in THY-CP medium [38% (vol/vol) THY medium and 62% (vol/vol) CP buffer containing 20 mM Tris-HCl pH 7.5, 0.1 M NaCl, 10 mM β-mercaptoethanol, 3 mM CaCl_2_] supplemented with 2 µM MnCl_2_ and indicated concentrations of recombinant WT (**A**), ∆His_3_Asp site (Zn) (**B**), ∆His_6_ site (**C**), or ∆His_3_Asp site/∆His_6_ site (**D**) mutant CP. After 6 h of incubation, cells were serially diluted, plated, and GAS CFUs were enumerated. Three biological replicates were grown on separate occasions, and the mean ± standard deviation is shown. *P* values (**P < 0.01, ***P < 0.001) were determined by comparison to respective WT GAS control and were derived from the Kruskal−Wallis test.

To investigate the contribution of Mn acquisition by MtsABC to GAS defense against Mn withholding by CP, we compared the sensitivity of GAS strains to CP by performing colony-forming unit (CFU) analyses. GAS strains were grown in THY-M in the presence or absence of increasing concentrations of CP, and cells collected at 6 h post-inoculation (hpi) were assessed for survival. All strains had similar growth characteristics in the absence of CP (Fig. 2A). However, when grown in the presence of CP, inactivation of *mtsC* (∆*mtsC*) resulted in increased sensitivity to CP, and the defective survival of the ∆*mtsC* mutant was reversed to WT-like phenotype in the *trans*-complemented strain (∆*mtsC:pDC-mtsC*) (Fig. 2A). Furthermore, the ∆*mtsC* mutant had WT-like survival in the presence of ∆His_3_Asp site/∆His_6_ site mutant CP that does not bind metals, indicating that the defective phenotype of the ∆*mtsC* mutant is due to metal sequestration by CP (Fig. 2D). These results indicate that MtsABC is critical for GAS survival in the presence of CP (Fig. 2A).

To delineate whether the increased sensitivity of the ∆*mtsC* mutant is due to Mn or Zn sequestration by CP, we performed growth experiments using recombinant CP mutant proteins with altered metal-binding properties. The ∆His_3_Asp site mutant CP in which Zn binding is impaired, but Mn binding is retained, and the ∆His_6_ site mutant CP with reduced Zn binding and defective Mn binding were used. Consistent with the role of MtsABC as an Mn importer, the ∆*mtsC* mutant was defective in survival during growth in the presence of Mn-binding ∆His_3_Asp site mutant CP (Fig. 2B). However, the ∆His_6_ site mutant CP did not impact the survival of the ∆*mtsC* mutant as it had WT-like survival phenotype in the presence of Mn-binding-deficient ∆His_6_ site mutant CP (Fig. 2C). Importantly, the *trans*-complemented strain (∆*mtsC:pDC-mtsC*) had WT-like CP resistance phenotype (Fig. 2). Collectively, these results demonstrate that MtsABC-mediated Mn acquisition aids GAS to mitigate CP-mediated Mn limitation and promotes bacterial survival in the presence of CP.

### MtsABC contributes to GAS oxidative stress defenses

**Fig 3:**
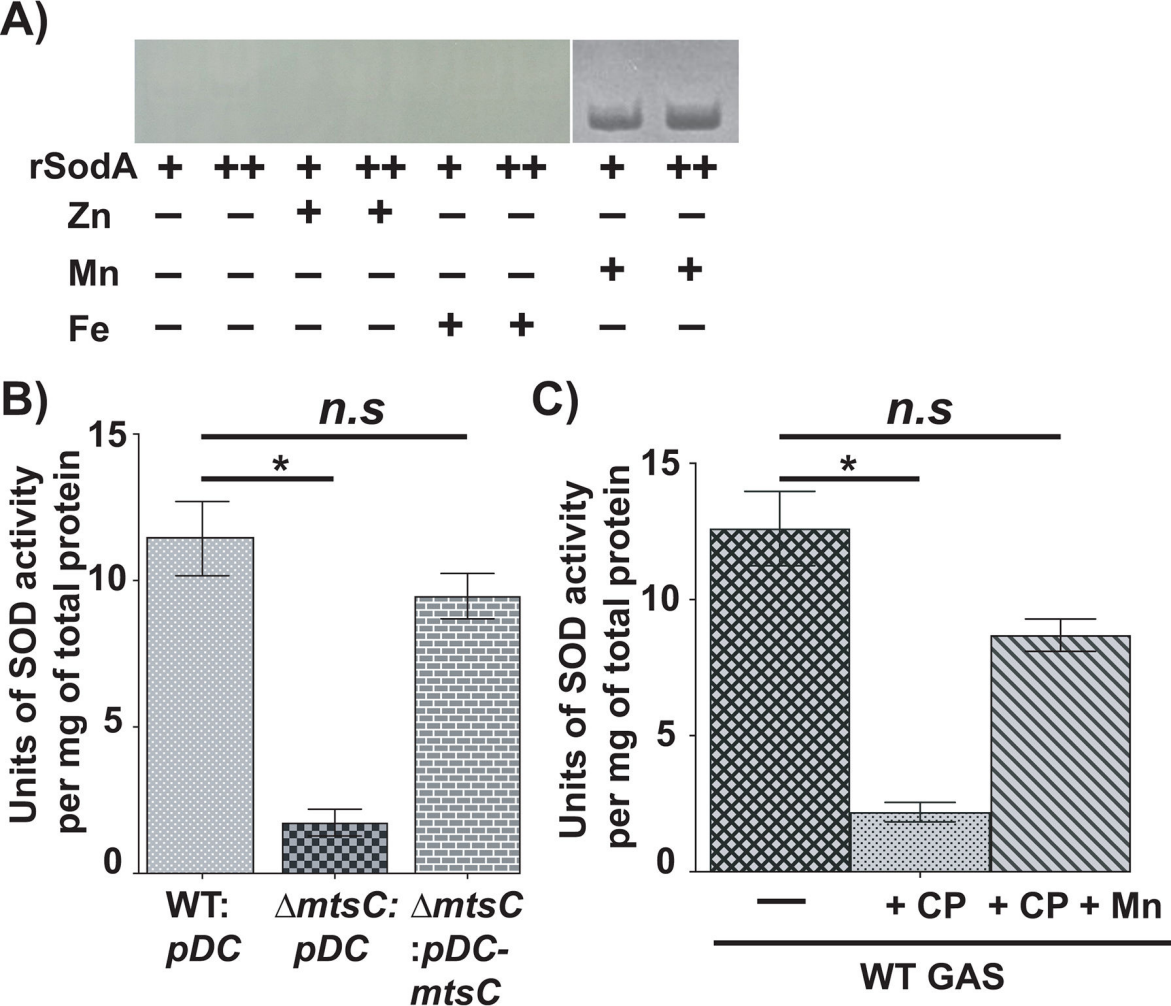
Mn-dependent activity of SodA is sensitive to Mn scarcity caused by *mtsC* inactivation or CP. (**A**) Native-PAGE analyses of recombinant SodA for SOD activity. Purified recombinant SodA was incubated with or without 5 µM of indicated metals, and the reaction mixture was resolved on a 10% native polyacrylamide gel. The presence of a band corresponding to SodA indicates SOD activity. (**B**) Indicated strains were grown in THY + CP buffer supplemented with 2 µM Mn to mid-exponential phase of growth (*A*_600_ ~1.0) and incubated with 2 mM paraquat for 15 min. Cell lysates were assessed for SOD activity. (**C**) The WT GAS was grown in THY + CP buffer supplemented with 2 µM Mn with or without 250 µg/mL of CP. To assess Mn-dependent restoration of SOD activity, 10 µM Mn was added. Cells were grown to mid-exponential phase of growth (*A*_600_ ~1.0) and incubated with 2 mM paraquat for 15 min. Cell lysates were assessed for SOD activity. *P* values (*P < 0.05) were determined by comparison to respective WT GAS control and were derived from the Kruskal–Wallis test.

The Mn sequestration by CP negatively impacts the detoxification activity of Mn-dependent bacterial superoxide dismutases (SOD), which results in increased bacterial sensitivity to oxidative stress, and reduced bacterial survival (6, 9). GAS encodes a sole Mn-dependent SodA that is critical for oxidative stress resistance and survival (30, 32). To test the hypothesis that the enzymatic activity of Mn-dependent SodA is sensitive to CP-dependent metal withholding, we purified recombinant SodA (rSodA) and tested the metal specificity of its catalytic activity by nitro blue tetrazolium assay. The purified rSodA did not exhibit activity in its non metallated form or in the presence of Fe or Zn (Fig. 3A). However, consistent with previous findings (30), the catalytic activity of rSodA was observed in the presence of Mn (Fig. 3A), indicating that SodA activity is Mn dependent.

To determine the significance of MtsABC-mediated Mn uptake for detoxification by SodA, we compared SodA activity among WT, ∆*mtsC*, and ∆*mtsC:pDC-mtsC* strains using an SOD activity kit. GAS was grown to the mid-exponential phase of growth, exposed to peroxide stress for 15 min, and assessed for intracellular SOD activity. Consistent with previous studies (30), the ∆*mtsC* mutant had significantly reduced SOD activity compared to WT GAS, whereas the *trans*-complemented strain had WT-like activity (Fig. 3B), indicating that Mn acquisition by MtsABC is critical for ROS detoxification by SOD.

Since CP sequesters Mn during infection, we assessed SOD activity in GAS grown in the presence of CP. GAS was grown in the presence or absence of CP to the mid-exponential phase of growth, incubated with H_2_O_2_ for 15 min, and cytosolic SOD activity was measured. The SOD activity was significantly reduced in CP-treated GAS growth relative to untreated growth (Fig. 3C). However, the impaired catalytic activity of SOD in the presence of CP was restored by the addition of Mn (Fig. 3C), indicating that the reduced intracellular SOD activity in the presence of CP is due to Mn scarcity. Collectively, these results demonstrate that MtsABC promotes Mn-dependent GAS antioxidant defenses during growth under Mn-limiting conditions caused by CP.

### MtsABC is critical for GAS survival in human saliva

**Fig 4:**
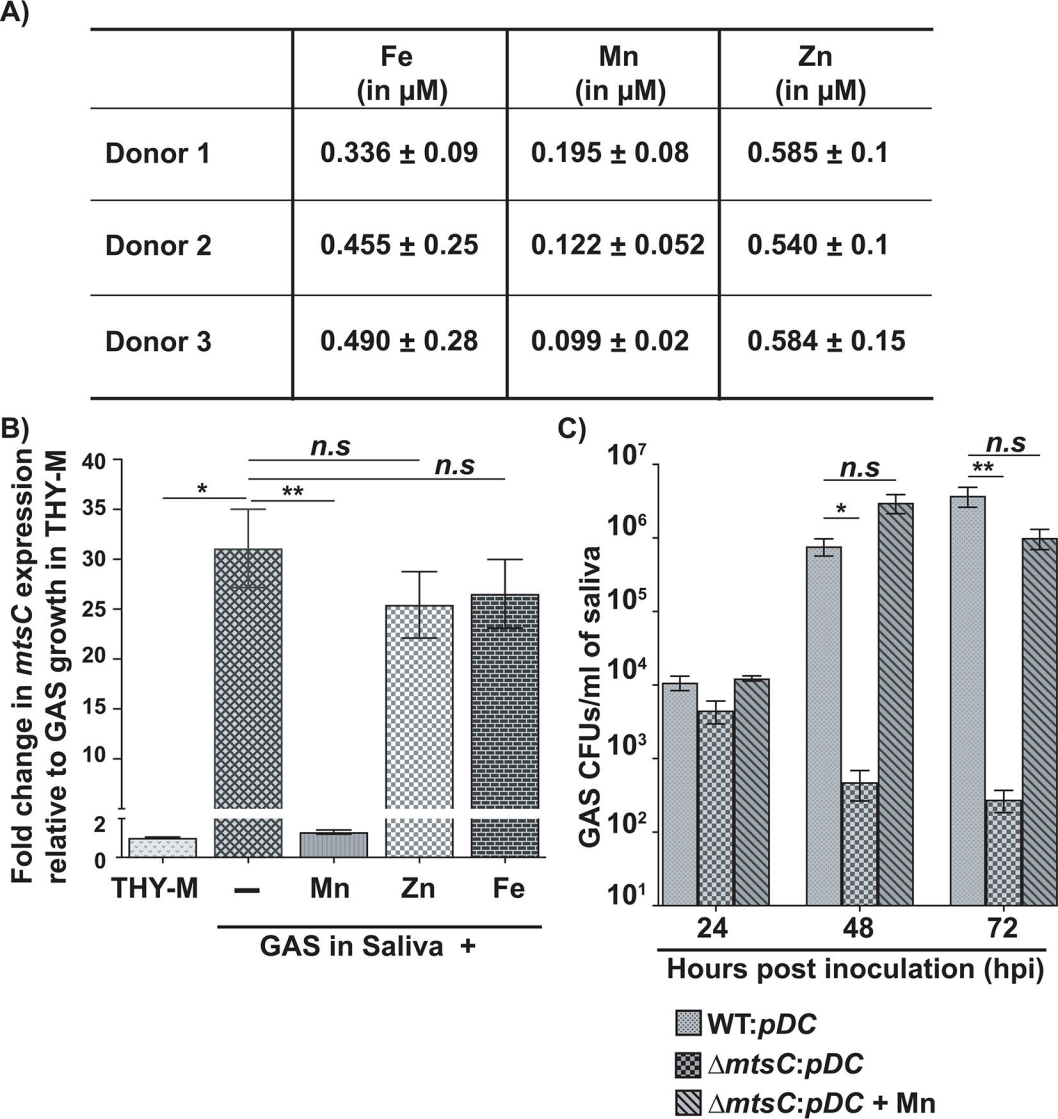
MtsABC is critical for GAS survival in human saliva *ex vivo*. (**A**) TXRF analyses of metal content in human saliva collected from three healthy donors. Samples were analyzed in triplicate, and the mean ± standard deviation is shown. (**B**) WT GAS was grown in saliva in the presence or absence of 2 µM of indicated metals. GAS grown in THY-M was used as the reference bacterial growth. Transcript levels of the *mtsC* was assessed using qRT-PCR. The fold change in transcript levels relative to reference is shown. Data graphed are mean ± standard deviation for three biological replicates grown on separate occasions. (**C**) The human saliva was inoculated with 10^3^ CFUs of each indicated GAS strain. Samples were collected at the indicated time points, serially diluted, plated, and GAS CFUs were enumerated. Three biological replicates grown on separate occasion were used, and the mean ± standard deviation is shown. *P* values (*P < 0.05 **P < 0.01) were determined by comparison to respective WT GAS control and were derived from Kruskal–Wallis test.

The human nasopharynx is the major port of GAS entry into the host, and pharyngitis is the most common form of GAS disease manifestations (33, 34). Although GAS colonizes the mucosal surfaces of oropharynx, GAS is released into the saliva during oropharyngeal colonization due to the shedding of host epithelia and epithelia-associated bacteria. Consistent with this, human saliva has a significant GAS load (>10^7^ CFUs/mL) during acute pharyngitis, and the presence of GAS in human saliva is critical for disease transmission between hosts via salivary aerosols (35–37). Given the significance of bacterial survival in human saliva for GAS pathogenesis, we investigated the contribution of MtsABC to GAS growth in human saliva *ex vivo*. We first assessed Fe, Mn, and Zn levels in human saliva collected from three healthy donors using TXRF analysis. The Fe, Mn, and Zn levels were comparable among samples, suggesting that minimal variations in metal levels exist in the saliva from different individuals (Fig. 4A). All three transition metals were present at sub-micromolar concentrations, and the Mn levels were relatively lower (~100–200 nM) compared to those of Fe and Zn (Fig. 4A). Given that the laboratory medium with an estimated 250 nM Mn represents Mn-limiting GAS growth conditions (14), these observations suggest that human saliva with scant levels of Mn represents a Mn-limiting host niche for GAS growth.

To test the hypothesis that GAS senses Mn limitation during growth in human saliva *ex vivo*, we measured *mtsC* transcript levels during GAS growth in human saliva by qRT-PCR. Using *mtsC* transcript levels in GAS grown in Mn replete THY-M as a reference, we assessed the alterations in *mtsC* expression during GAS growth in saliva. Our results show that GAS mounts significant induction (~30-fold increase) of *mtsC* expression during growth in saliva compared to THY-M, indicating that GAS senses Mn scarcity in saliva (Fig. 4B). To ensure that the observed increase in *mtsC* expression is specific for sensing of Mn limitation by GAS, we assessed *mtsC* expression during GAS growth in saliva supplemented with different metals. Only the addition of Mn to saliva abolished *mtsC* upregulation, whereas Fe or Zn supplementation failed to reverse the induction of *mtsC* expression (Fig. 4B). Together, these data show that GAS employs MtsABC during growth in Mn-sparse human saliva and suggest that Mn uptake by MtsABC is critical for GAS survival in human saliva.

To determine the significance of MtsABC to GAS survival in human saliva *ex vivo*, we compared the growth of WT and the ∆*mtsC* mutant in saliva. GAS reached a high population density (>10^6^ CFUs/mL) over 3 days in saliva *ex vivo* (Fig. 4C). However, the ∆*mtsC* mutant showed significant survival defect in saliva compared to WT GAS, and a gradual clearance was observed at 48 and 72 hpi (Fig. 4C). The defective survival of the ∆*mtsC* mutant in saliva was restored to WT levels by supplementation with Mn (Fig. 4C), indicating that Mn acquisition by MtsABC is critical for GAS survival in human saliva.

### MtsABC is critical for CP resistance *in vivo* and GAS virulence

**Fig 5:**
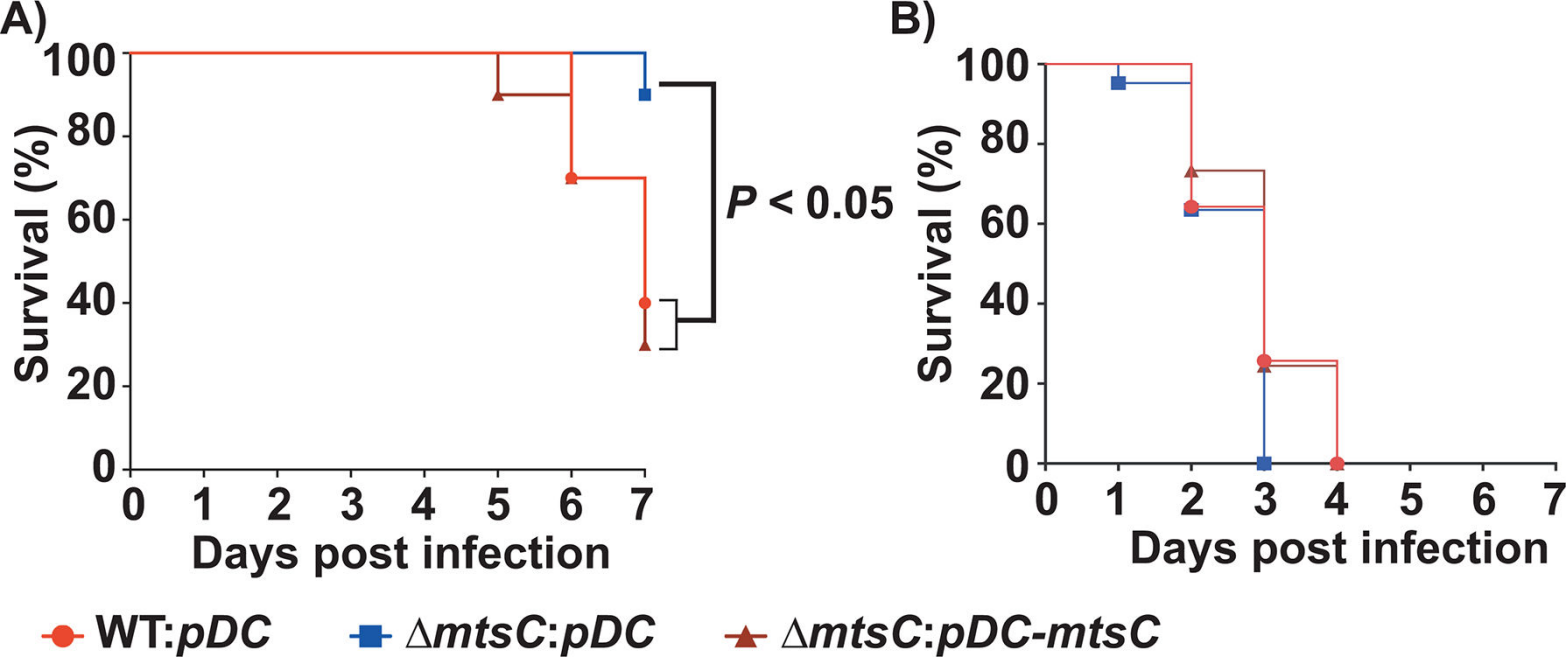
Gene encoding *mtsC* is critical for GAS resistance against CP-mediated Mn limitation *in vivo* and contributes significantly to GAS virulence. The *S100a9^+/+^* (WT) (**A**) or *S100a9^−/−^* (**B**) mice (*n = 10* per group) were infected intramuscularly with 1 × 10^8^ CFUs of each indicated bacterial strain. Kaplan–Meier survival curve with *P-*values derived by log-rank test is shown.

Although it was previously shown that MtsABC is critical for GAS virulence in mouse models of invasive infection (27, 30), the role of MtsABC in GAS resistance to CP *in vivo* remains unknown. Thus, we hypothesized that MtsABC contributes significantly to GAS pathogenesis but is dispensable for virulence in mice lacking CP. To test this hypothesis, we assessed the virulence traits of WT, ∆*mtsC*, and ∆*mtsC:pDC-mtsC* strains in an intramuscular mouse model of invasive infection. Mice were inoculated intramuscularly with 10^8^ CFUs of each GAS strain and observed for near mortality. The ∆*mtsC* mutant was significantly attenuated for GAS virulence compared to the WT and *trans*-complemented strains (*P <* 0.05 when comparing ∆*mtsC* mutant with WT) (Fig. 5A), indicating that MtsABC is critical for GAS virulence.

To determine the role of MtsABC in CP resistance *in vivo*, we compared the virulence phenotype of GAS strains using *S100 a9^−/^*^−^ mice. Contrary to the observations in WT mice, the virulence phenotype of ∆*mtsC* mutant was similar to that of WT GAS in *S100 a9^−/−^* mice (*P <* 0.05 when comparing ∆*mtsC* mutant with WT), indicating that MtsABC is not required for GAS virulence in the absence of host-mediated Mn sequestration (Fig. 5B). Together, the results from animal infection studies show that Mn acquisition by MtsABC contributes to GAS evasion of CP-mediated metal limitation *in vivo* and GAS pathogenesis.

## DISCUSSION

Previously, we demonstrated that the host imposes Zn limitation on GAS during infection, and the GAS gene products critical for survival during Zn scarcity participate in the defense against host nutritional immune mechanisms (12, 13). In this study, we investigated the interplay between host-mediated Mn withholding and GAS counterstrategies and its impact on GAS pathogenesis. Our results demonstrate that the Mn importer MtsABC is a major component of GAS defenses that aids evasion of CP-imposed Mn sequestration and survival in the host. We show that CP imposes Mn starvation on GAS during growth *in vitro,* and GAS deploys the Mn importer MtsABC in response to Mn withholding by CP (Fig. 1). Mn acquisition by MtsABC is critical for the cytosolic Mn levels, GAS CP resistance (Fig. 2), and intracellular ROS detoxification activity of Mn-dependent cytosolic SodA (Fig. 3). Furthermore, we found that human saliva, the major GAS reservoir in the host, is a Mn-scant body fluid, and MtsABC plays a crucial role in GAS survival in saliva *ex vivo* (Fig. 4). Finally, animal infection studies demonstrated that MtsABC contributes significantly to GAS pathogenesis in an invasive mouse model of infection. However, Mn acquisition by MtsABC is dispensable for GAS virulence in mice lacking CP (Fig. 5), emphasizing the direct competition for Mn between host-derived CP and GAS Mn importer MtsABC during infection.

Typically, bacterial pathogens encode multiple Mn uptake systems that belong to different families of metal transporters, and the functionally redundant transporters contribute collectively to bacterial Mn acquisition and virulence (9, 38–43). In *Enterococcus faecalis*, three different Mn importers participate in Mn acquisition, and simultaneous inactivation of two Mn importers is required for CP sensitivity and virulence (43). Similarly, *Streptococcus mutans* also encodes two Mn importers, and inactivation of individual importers did not affect the bacterial survival in the presence of CP, indicating the functional redundancy of the two Mn importers (38). Finally, *Salmonella typhimurium* has three Mn uptake systems belonging to distinct families of transporters, which contribute collectively to bacterial CP resistance (9). Contrary to these observations, MtsABC is the only known Mn importer encoded in the GAS genome. Thus, it is not surprising that inactivation of *mtsABC* significantly impaired Mn uptake (Fig. 1B), GAS survival in the presence of CP (Fig. 2), and attenuated bacterial virulence (Fig. 5).

Despite the known significance of MtsABC to GAS virulence (27–30, 44), the mechanism by which MtsABC contributes to GAS pathogenesis remains undefined. Our findings elucidate two niche-specific roles for MtsABC-mediated Mn acquisition in bacterial survival *in vivo* and GAS virulence. The human saliva is a major GAS reservoir, and GAS survival in the saliva is critical for pharyngitis development and disease transmission (36, 37). We show that saliva from healthy subjects is a naturally Mn-scant body fluid, and MtsABC contributes significantly to GAS persistence in the human saliva *ex vivo*, even in the absence of CP-mediated Mn sequestration (Fig. 4). Thus, it is likely that MtsABC plays a significant role in GAS colonization of the oropharynx and the dissemination of GAS infection to susceptible individuals. Additional investigations are required to determine the significance of Mn acquisition by MtsABC in oropharyngeal GAS colonization and pharyngitis development. On the other hand, during invasive infections, GAS is engaged in competition with CP for Mn in infected abscesses, and the high-affinity Mn uptake by MtsABC promotes GAS virulence by negating the impact of host-imposed Mn limitation (Fig. 5).

The identity of the specific metal transported by MtsABC remains unclear. Earlier studies suggested that MtsABC transports Fe, Zn, and Cu (44), whereas biophysical characterization of the extracellular subunit MtsA showed that MtsA binds Fe preferentially over Mn (28, 29). However, recent work has indicated that MtsABC is specifically involved in Mn acquisition (30). Our results show that the inactivation of *mtsABC* caused a more pronounced reduction in intracellular Mn concentration, but it also caused a significant reduction in Fe levels (Fig. 1B), suggesting a role for MtsABC in Fe uptake. Interestingly, it has been shown that CP binds Fe using the Mn-binding site with the His_6_ motif and imposes Fe limitation on various pathogens during growth *in vitro* (45, 46). Thus, it is possible that MtsABC is also involved in Fe acquisition and GAS resistance to CP-mediated Fe limitation. Further investigations are required to delineate the physiological relevance of Fe acquisition by MtsABC, Fe sequestration by CP, and the effect of interplay between MtsABC and CP for Fe on GAS pathogenesis.

However, several layers of evidence support a Mn-specific transport function and suggest a non-specific, promiscuous import of Fe by MtsABC. First, we previously showed that GAS activates *mtsABC* expression specifically during Mn scarcity, and such modulation of *mtsABC* expression does not occur in response to fluctuations in Fe levels (14). Second, similar Mn-specific, Fe-insensitive regulation of *mtsABC* expression was observed during GAS growth in human saliva (Fig. 4B). Finally, our prior studies also showed that the transcription regulator, MtsR, that directly controls *mtsABC* expression is a Mn-sensing repressor and unresponsive to Fe (14). MtsR senses Mn sufficiency, and the Mn-bound MtsR downregulates *mtsABC* expression by engaging in sequence-specific high affinity interactions with *mtsABC* promoter (14). Conversely, during Mn scarcity, the metal-free MtsR dissociates from *mtsABC* promoter and relieves the repression of *mtsABC* expression (14). We showed that the high-affinity interactions between MtsR and *mtsABC* promoter is Mn specific and insensitive to Fe. Given that GAS deploys MtsABC specifically during growth in Mn deficiency, these observations lend support to our model that MtsABC confers protection against CP-mediated Mn limitation.

During infection, pathogenic bacteria encounter neutrophil-mediated oxidative burst, which involves the release of toxic ROS and bacterial killing by the oxidation of macromolecules (47–49). The Mn-dependent bacterial SODs play a protective role in negating ROS toxicity by enzymatically detoxifying cytosolic superoxide (50, 51). A well-documented downstream molecular event of CP-mediated Mn sequestration is the impairment of superoxide detoxification function of Mn-dependent SODs encoded by several human pathogens (6, 9, 52). GAS also encodes a Mn-dependent SodA, which is critical for GAS survival during oxidative stress (27, 30, 32). We found that the detoxification activity of SodA is sensitive to CP-mediated Mn sequestration, and Mn acquisition by MtsABC promotes the detoxification activity of SodA (Fig. 3). These findings further emphasize the significance of MtsABC to GAS survival in the host and pathogenesis.

In summary, the results presented here demonstrate that nutrient withholding is a key defense mechanism employed by the host to limit the proliferation of a human pathogen. However, we found that the pathogen employs a molecular arsenal to overcome host defenses and survive in the host. The elucidation of bacterial counterstrategies that are critical for their competition against host defenses and bacterial survival is crucial to the molecular understanding of host–pathogen interactions during infection and identify potential translational targets to combat human infections.

## MATERIALS AND METHODS

### Bacterial strains, plasmids, and growth conditions

The bacterial strains and plasmids used in this study are listed in Table S1. Strain MGAS10870 is a representative of serotype M3 strains that cause invasive infections (53). The whole genome of MGAS10870 has been fully sequenced and has wild-type sequences for all major regulatory genes (53). We used *E. coli* DH5α as the host for plasmid cloning, whereas *E. coli* BL21(DE3) was used for recombinant protein overexpression. GAS strains were routinely grown in Todd−Hewitt broth containing 0.2% (wt/vol) yeast extract (THY; Difco) or Trypticase soy agar containing 5% sheep blood (bovine serum albumin; Becton, Dickinson). For growth studies in the presence of CP, THY-CP medium was prepared by adding 38% (vol/vol) THY to 62% (vol/vol) CP medium [20  mM Tris HCl (pH 7.5), 100  mM NaCl, 10  mM β-mercaptoethanol, and 3  mM CaCl_2_]. *E. coli* strains DH5α and BL21(DE3) were grown in lysogeny broth (LB; Teknova). Overnight cultures of GAS were inoculated in fresh medium with an initial absorption of 0.03 at *A*_600_. Bacterial growth was monitored by measuring the optical density at *A*_600_ with a microplate reader. Chloramphenicol and ampicillin were added to the cultures to a final concentration of 5 and 80  µg/mL, respectively, when required.

### Construction of *mtsC*-inactivated and *trans*-complemented strains

Isogenic strains containing gene inactivation of the entire coding region of interest were generated as previously described (54). Briefly, a PCR fragment with the entire coding region of interest to be deleted was generated using a two-step PCR process. In step 1, the PCR fragment was generated by amplifying the 5′ or 3′ flanking region of the gene of interest. In step 2, a single PCR fragment containing the fusion of the 5′ and 3′ flanking regions without the gene of interest was generated and subsequently cloned into the multi-cloning site of the temperature-sensitive plasmid pJL1005 (55). The resultant plasmids were introduced into GAS by electroporation, and colonies with plasmid incorporated into the GAS chromosome were selected for subsequent plasmid curing. The presence of the desired mutations and the absence of spurious mutations were confirmed by DNA sequencing.

To generate the *trans*-complemented strain ∆*mtsC:pDC-mtsC*, the coding region of the full-length *mtsC* gene, along with the putative *mtsABC* promoter, was cloned into the *E. coli*-GAS shuttle vector *pDC123* (55). Using the primers listed in Table S2, the corresponding fragments were amplified by PCR from GAS genomic DNA and ligated into a digested vector to generate the complementation plasmid, *pDC-mtsC*. The insert was verified by DNA sequencing, and *pDC-mtsC* was electroporated into the strain MGAS10870-Δ*mtsC.* The primers used to generate isogenic mutant and the *trans*-complemented strains are listed in Table S2.

### Metal content analyses using total-reflection X-ray fluorescence (TXRF) analysis

GAS strains were grown in THY-M to mid-exponential phase of growth (*A*_600_ ~1.0). Cells were harvested, cell pellets were washed twice with chelexed phosphate-buffered saline (PBS) and once with chelexed PBS supplemented with 1 mM nitrilotriacetic acid. Cells were washed again with chelexed PBS and resuspended in chelexed PBS. Cells were lysed by fast prep, and cytosolic contents were isolated by centrifugation at 15,000 rpm for 15 min at 4°C. The clarified supernatant was used for metal content analyses using TXRF. Three biological replicates of each strain grown on separate occasions were used for metal content analyses. TXRF analyses were performed as described previously (56–58). Each biological replicate was prepared twice and measured with a Bruker PicoFox S2 spectrometer. Gallium at a concentration of 200 µg/L in distilled water was used as an internal standard; 7 µL of the standard was mixed with 7 µL of sample. Of this mixture, 10 µL was transferred to a siliconized quartz sample carrier and dried with the help of a hot plate. Each disc was measured for 1,000 s, and the results were analyzed with the software provided with the instrument by Bruker, Spectra version 7.8.2.0. The results of the two measurements were averaged and used for further data analysis.

### Recombinant CP purification

Overexpression and purification of recombinant human WT or mutant calprotectin (CP) were carried out as previously described (13). Overnight cultures of BL21(DE3) containing the coding sequences of *S100a8* or *S100a9* in plasmid pET15b were diluted 1:50 in fresh LB medium and grown at 37°C until the *A*_600_ reached 0.4 to 0.6. Protein overexpression was induced by the addition of 1  mM isopropyl β-d-1-thiogalactopyranoside (IPTG), and cells were grown at 37°C for an additional 4 h. Equal amounts of cell pellets (by weight) containing *S100a8* and *S100a9* overexpression plasmids were suspended in buffer A [20  mM Tris HCl (pH 8.0), 0.1 M NaCl, 10  mM β-mercaptoethanol, 1  mM EDTA, and 0.5% Triton X-100] supplemented with a protease inhibitor pellet (Roche). Cells were lysed with a cell lyser (Microfluidics), and inclusion bodies containing S100A8 and S100A9 proteins were fractionated by centrifugation at 21,000  ×  *g* for 30 min. The pellet was resolubilized by suspending it in buffer containing 50  mM Tris HCl (pH 8.0), 100  mM NaCl, 10  mM β-mercaptoethanol, and 4 M guanidine hydrochloride. Refolding of the proteins was achieved by overnight dialysis using a 10-kDa cutoff membrane against the base buffer containing 20  mM HEPES (pH 8.0). After three rounds of dialysis, the final dialyzed sample was centrifuged at 21,000  ×  *g* for 30  min and filtered with a 0.22-µm syringe filter. The S100A8/S100A9 heterodimer was separated by ion-exchange chromatography using a Mono-Q column (GE Lifesciences) preequilibrated with base buffer [20 mM HEPES (pH 8.0), 10 mM β-mercaptoethanol] and eluted using a salt gradient of 0 to 300  mM NaCl. The sample was further purified by size exclusion chromatography using Superdex 26/600 200 kDa (GE Lifesciences). Finally, the metal-free form of the S100A8/S100A9 heterodimer was prepared with a two-step dialysis as follows: first, against storage buffer containing 20  mM HEPES (pH 8.0), 100  mM NaCl, 10  mM β-mercaptoethanol, and 10  mM EDTA and, second, against chelexed storage buffer without EDTA. The S100A8/S100A9 heterodimer was concentrated using a YM-10 filter to a final concentration of 10 mg/mL, and flash frozen aliquots were stored at –80°C until used.

### GAS growth studies with CP

A GAS inoculum of 10^5^ CFUs/mL was inoculated into THY-M broth supplemented with CP buffer. The recombinant purified CP was added to the starter culture. After 6 h of incubation, cells were collected, serially diluted, plated, and subjected to bacterial survival analyses by enumerating CFUs. Samples were analyzed in triplicate, and at least two different CP preparations were used.

### Transcript level analyses

GAS strains were grown under the indicated growth conditions and incubated with two volumes of RNAprotect (Qiagen) for 10 min at room temperature. Cells were harvested by centrifugation, and the cell pellets were snap-frozen in liquid nitrogen. RNA isolation and purification were performed using RNeasy kit (Qiagen) according to the manufacturer’s protocol. The concentration of RNA was measured using a NanoDrop 8000 instrument (Thermo Fisher Scientific). cDNA was synthesized from 2 µg of total RNA using superscript III (Invitrogen), and quantitative PCR (qPCR) was performed using SYBR green Q-PCR master mix (GenDEPOT) with an AB1 7500 fast system (Applied Biosystems). The specificity of the reaction was verified with melt curve analysis. Comparison of transcript levels was performed using the threshold cycle (Δ*C_T_*) method of analysis using *tufA* as the endogenous control gene (59). The primers used for qRT-PCR) are listed in Table S2.

### Recombinant SodA purification

Overnight cultures of BL21(DE3) containing the coding sequences of GAS *sodA* in plasmid pET21b were diluted 1:50 in fresh LB medium and grown at 37°C until the *A*_600_ reached 0.4 to 0.6. Protein overexpression was induced by the addition of 1  mM IPTG, and cells were grown at 30°C for an additional 6 h. Cell pellets were suspended in buffer A [20  mM Tris HCl (pH 8.5), 0.1 M NaCl, and 1  mM tris(2-carboxyethyl)phosphine] supplemented with a protease inhibitor pellet (Roche). Cells were lysed with a cell lyser (Microfluidics), and cell lysates were separated by centrifugation at 21,000  ×  *g* for 30  min. The hexa-histidine-tagged SodA was purified by Ni-NTA affinity chromatography using 0–250 mM imidazole gradient. The eluted fractions were pooled, concentrated using a 10-kDa cut-off membrane filter, and purified using size exclusion chromatography. The purified SodA was dialyzed against storage buffer (20 mM Tris pH 8.5, 0.1 M NaCl, and 5% glycerol) containing 10 mM EDTA to remove residual metals bound to SodA. The protein purified to near homogeneity (>95% purity) was further dialyzed against chelexed storage buffer without EDTA twice and stored at −80°C.

### SOD assay by native PAGE

Purified recombinant SodA was added to chelexed reaction buffer (20 mM Tris pH 8.5, 0.1 M NaCl) to a final concentration of 4 µg and incubated with or without 5 µM of indicated metals. Samples were incubated at room temperature for 10 min and loaded on a 10% native polyacrylamide gel. Samples were resolved at 150 V for 15 min in Tris-Glycine running buffer. The resolved gels were soaked in riboflavin–nitroblue tetrazolium mixture for 15 min, and gels were visualized using a Bio-Rad gel imager.

The SOD activity was assessed by the presence or absence of a white band corresponding to SodA (60).

### SOD activity assay

SOD activity in GAS strains with or without CP was determined by commercially available SOD-WST kit (Sigma Aldrich) (61). Briefly, 300 µL of cells (approximately 25–100 U mL^−1^ SOD) were grown in the presence or absence of CP. When cells reached mid-exponential phase of growth, paraquat was added to a final concentration of 2 mM, and cells were incubated for 15 min at 37°C. Cells were harvested, washed with 50 mM HEPES pH 7.4, and resuspended in 150 µL of 50 mM HEPES pH 7.4. Cell lysates were collected, and total protein concentrations of the samples were determined using Bradford assay. The SOD activity was assayed as per the manufacturers’ instructions.

### Collection of human saliva

Saliva from adult volunteers was collected on ice under a protocol approved by the Institutional Review Board at Houston Methodist Research Institute (approval number Pro00003833) using the method described previously with minor modifications (30). The saliva was clarified by centrifugation at 23,000 × *g* for 1 h, followed by filtration through a 0.22-μm-pore-size membrane filter (Corning, NY). Pooled saliva was stored frozen at −20°C. Saliva from at least four donors was pooled to minimize the potential effects of donor variation.

### Bacterial survival studies in saliva

The ability of GAS strains to grow and persist in human saliva was evaluated as described previously (62, 63). Briefly, human saliva was collected from healthy volunteers and pooled as described above. GAS was grown overnight in Todd–Hewitt broth supplemented with 0.2% yeast extract (THY; BD Biosciences, Sparks, MD), diluted 1:100 with fresh THY, and grown to the growth phase indicated above. The bacterial cells were pelleted, washed twice with sterile PBS, and suspended in saliva at ~1 × 10^3^ CFU/mL. Aliquots were removed at the time points indicated in the figures. Samples were serially diluted 10-fold in sterile PBS and plated in duplicate on trypticase –soy agar plates supplemented with 5% sheep blood (BD Biosciences). The plates were incubated overnight, and colonies were counted to determine the number of CFU. All incubations were at 37°C with 5% CO_2_. Each experiment was performed in triplicate on three separate occasions.

### Animal infection studies

The virulence of the GAS strains was assessed using an intramuscular mouse model of infection. Ten 7- to 8-week-old WT C57 (*S100a9*^+/+^) *or S100a9*^−/−^ mice were inoculated in the right hindlimb with 1  ×  10^8^ CFU of each strain and monitored for near mortality. Results were graphically displayed as a Kaplan–Meier survival curve and analyzed using the log-rank test.

## SUPPLEMENTAL MATERIAL

Supplemental material iai.00077-24-s0001.pdf Tables S1 and S2; Figure S1.

ASM does not own the copyrights to Supplemental Material that may be linked to, or accessed through, an article. The authors have granted ASM a non-exclusive, world-wide license to publish the Supplemental Material files. Please contact the corresponding author directly for reuse.
