# Supplementary material for: Manganese uptake by MtsABC contributes to the pathogenesis of human pathogen group A streptococcus by resisting host nutritional immune defenses
Source: Infect Immun. 2024 Jun 13;92(7):e00077-24. doi: 10.1128/iai.00077-24 (PMC11238556; doi:10.1128/iai.00077-24)
Supplement: Supplemental material — Tables S1 and S2; Figure S1. [file iai.00077-24-s0001.pdf]

Supplementary table S1. Bacterial strains and plasmids used in this study

Strain or Plasmid	Description	Reference
**Strains**		
WT	Invasive isolate MGAS10870, serotype M3	(1)
WT:*pDC*	WT with empty vector *pDC123*, Cm^+	(2)
Δ*mtsC*	Isogenic mutant strain that has the in-frame deletion of *mtsC* in parental serotype MGAS10870	This study
Δ*mtsC*:*pDC*	MGAS10870 Δ*mtsC* with empty vector *pDC123*, Cm^+	This study
Δ*mtsC:pDC-mtsC*	*Trans*-complemented strain derived introducing plasmid *pDC* containing *mtsC* promoter along with the *mtsC* gene into the Δ*mtsC* mutant strain	This study
E. coli DH5α	Host strain for cloning purposes	
E. coli BL21 (DE3)	Host strain for protein overexpression, *F-, ompT, hsdSB(Rb-Mb-), gal(λ c I 857, ind1, Sam7, nin5, lacUV-T7 gene1), dcm(DE3)*	
**Plasmids**		
pJL	Shuttle plasmid capable of replication in *Escherichia* coli but suicide vector in GAS, Cm^+<br>Used to generate isoallelic GAS mutants	(3)
pDC123	Low-copy number plasmid capable of replication in *Escherichia* coli and GAS, Cm^+<br>Used to trans-complement isoallelic GAS mutants	(4)
pET15b	Overexpression vector used for the production of tag-free S100A8 and S100A9, Amp resistant	(5)
pET21b	Overexpression vector used for the production of recombinant SodA, Amp resistant	

Supplementary table S2. Primers used in this study

Primer	Sequence 5' – 3'	Purpose
ΔmtsC-A	AGGTCACGTAACGGTTGATG	5' primer for 5' region of *mtsC* to delete *mtsC*
ΔmtsC-B	GCGAAAAGTTTAAGGTGT TCATAGACATTTACACCATCTC	3' primer for 5' region of *mtsC* to delete *mtsC*
ΔmtsC-C	GAGATGGTGTAAATGACACCTTAAACTTTT CGC	5' primer for 3' region of *mtsC* to delete *mtsC*
ΔmtsC-D	CAAAGGAGATGGCACAGGTGGTGAATCG	3' primer for 3' region of *mtsC* to clone into *pJL* to delete *mtsC*
pDC-mtsC Fwd	GTCATATCGTTTGGCCTTTCTC	5' primer for 5' region of *mtsC promoter* to clone into *pDC*
pDC-mtsC Rev	TAAAGCGAAAAGTTTAAGGTG	3' primer for 3' region of *mtsC gene* to clone into *pDC*
mtsC qRTFwd	CAACTCGTCACTATGCGCACAT	5' primer for *mtsC* qRT-PCR
mtsC qRTRev	GAGCGGCACCAGTGATCAT	3' primer for *mtsC* qRT-PCR
tufA qRTFwd	CAACTCGTCACTATGCGCACAT	5' primer for *tufA* qRT-PCR
tufA qRTRev	GAGCGGCACCAGTGATCAT	3' primer for *tufA* qRT-PCR

References

1. Beres SB*, et al.* (2010) Molecular complexity of successive bacterial epidemics deconvoluted by comparative pathogenomics. *Proceedings of the National Academy of Sciences* 107(9):4371-4376.
2. Shelburne III SA*, et al.* (2011) An amino-terminal signal peptide of Vfr protein negatively influences RopB-dependent SpeB expression and attenuates virulence in *Streptococcus pyogenes*. *Mol. Microbiol.* 82(6):1481-1495.
3. Li J, Kasper DL, Ausubel FM, Rosner B, & Michel JL (1997) Inactivation of the α C protein antigen gene, bca, by a novel shuttle/suicide vector results in attenuation of virulence and immunity in group B Streptococcus. *Proceedings of the National Academy of Sciences* 94(24):13251-13256.

4. Chaffin D & Rubens C (1998) Blue/white screening of recombinant plasmids in Gram-positive bacteria by interruption of alkaline phosphatase gene (*phoZ*) expression. *Gene* 219(1-2):91-99.
5. Makthal N*, et al.* (2017) A Critical Role of Zinc Importer AdcABC in Group A Streptococcus-Host Interactions During Infection and Its Implications for Vaccine Development. *EBioMedicine* 21:131-141.

Supplementary figure S1

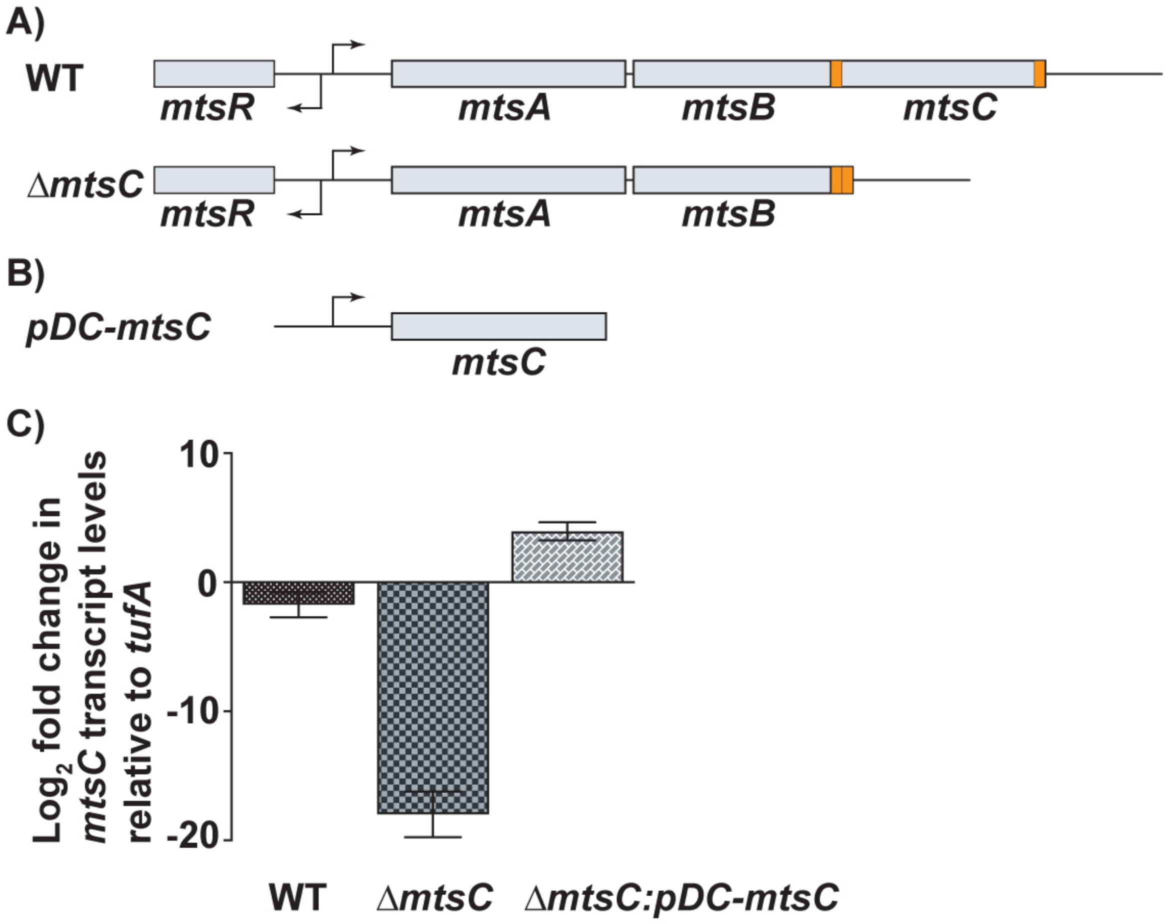

Supplementary figure S1. Schematics of the *mtsC* inactivated (Δ*mtsC*) strain and trans-complementation plasmid (*pDC-mtsC*) used in this study. A) Genetic arrangements of each gene in WT GAS (top) and mutant strain (bottom) are shown. The *mtsC* gene was inactivated by deleting the internal region of *mtsC* coding region. A large fragment in the central part of each gene (colored in grey) was removed by fusing two smaller fragments at either ends of the gene of interest (colored in orange). The arrows above and below the line indicate the transcription start sites of the indicated genes. **B)** The schematics of *trans*-complementation plasmid *pDC-mtsC* that has the *mtsA* promoter

(bent arrow) region fused with the coding region of *mtsC*. **C)** The *mtsC* transcript level analyses of the indicated strains by qRT-PCR.
